# Withdrawn as duplicate: A new year, a new era: Romania and Bulgaria join the European Union

**DOI:** 10.1093/eurpub/ckm021

**Published:** 2007-01-01

**Authors:** Martin McKee, Dina Balabanova, Andreea Steriu

**Affiliations:** European Centre on Health of Societies in Transition, London School of Hygiene and Tropical Medicine, Keppel Street, London WC1E 7HT, UK; Department of Home Affairs, Crookhall House, Demesne Road, Douglas, IM1 3QA, Isle of Man; Department of Home Affairs, Crookhall House, Demesne Road, Douglas, IM1 3QA, Isle of Man

This article has been withdrawn due to a publisher error that caused the article to be duplicated. The definitive version of this article is published under https://doi.org/10.1093/eurpub/ckm012.

